# Mitochondrial Amount Determines Doxorubicin‐Induced Cardiotoxicity in Cardiomyocytes

**DOI:** 10.1002/advs.202412017

**Published:** 2025-02-07

**Authors:** Weiyao Xiong, Bin Li, Jianan Pan, Dongjiu Li, Haihua Yuan, Xin Wan, Yanjie Zhang, Lijun Fu, Junfeng Zhang, Ming Lei, Alex Chia Yu Chang

**Affiliations:** ^1^ Department of Cardiology Shanghai Ninth People's Hospital Shanghai Jiao Tong University School of Medicine Shanghai 200011 China; ^2^ Shanghai Institute of Precision Medicine Shanghai Ninth People's Hospital Shanghai Jiao Tong University School of Medicine Shanghai 200125 China; ^3^ Department of Oncology Shanghai Ninth People's Hospital Shanghai Jiao Tong University School of Medicine Shanghai 200011 China; ^4^ Department of Cardiology Shanghai Children's Medical Centre Shanghai Jiao Tong University School of Medicine Shanghai 200127 China

**Keywords:** cGAS‐STING, doxorubicin induced cardiotoxicity, mitochondrial amount, mitochondrial transplantation, senescence

## Abstract

Doxorubicin, an anthracycline commonly used for treating cancer patients, is known for its cardiotoxic side‐effects. Although dose‐dependent, but susceptibility remains variable among patients, and childhood‐exposure‐adult‐onset remains challenging. Besides topoisomerase toxicity, Doxorubicin is also toxic to the mitochondria yet the underlying late onset mechanism remains elusive. Here, it is observed that the mitochondrial copy number in PBMCs of patients treated with anthracycline chemotherapy is negatively correlated with the change in plasma BNP levels after treatment. Isogenic hiPSC‐CMs are generated with high, norm, and low mitochondrial copy numbers using mitochondrial transplantation and the YFP‐Parkin system. Remarkably, lower mitochondria copy number translates to lower IC_50_, suggesting increased susceptibility. Mitochondria supplementation by intramyocardial injection prevents doxorubicin induced heart failure. Mechanistically, doxorubicin treatment leads to mPTP opening and mitochondrial DNA (mtDNA) leakage. This mtDNA leakage event activates the cGAS‐STING pathway and drives inflammation and myocardial senescence. Cardiomyocyte‐specific knockout of Sting (Myh6‐Cre/Sting^flox/flox^; Sting^CKO^) and over expression of mitochondrial tagged DNase1 in mice partially rescue doxorubicin‐induced cardiac dysfunction. In conclusion, the work establishes a negative correlation between cardiomyocyte mitochondrial copy number and doxorubicin toxicity. Molecularly, it is demonstrated that mtDNA leakage activates cGAS‐STING pathway and accelerates myocardial dysfunction. These insights offer new co‐administration strategies for cancer patients.

## Graphical abstract

Using human‐induced pluripotent stem cell‐derived cardiomyocytes (hiPSC‐CMs), it is demonstrated that mitochondrial abundance dictates sensitivity to DIC. Mechanistically, doxorubicin treatment elevates ROS, leading to the opening of the mitochondrial permeability transition pore (mPTP), mitochondrial DNA (mtDNA) leakage, and activation of the cGAS‐STING pathway. Through three distinct rescue strategies, it is established that inhibiting the mtDNA‐cGAS‐STING signaling axis effectively mitigates doxorubicin‐induced cardiotoxicity.

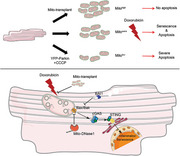

## 1 Introduction

Doxorubicin (Dox), a clinically used anthracycline with broad‐spectrum antitumor activity, is associated with severe cardiotoxicity, that leads to acute or late‐onset heart failure.^[^ ^1^ ^]^ It has been demonstrated that doxorubicin can target topoisomerase IIβ to increase reactive oxygen species, drive mitochondrial dysfunction, and result in myocardial loss and heart failure.^[^ ^2^ ^]^ Clinically, doxorubicin‐induced cardiomyopathy (DIC) is dose‐cumulative dependent and can occur long after treatment. When the cumulative doxorubicin dose reaches 600 mg m^−2^ risk of DIC is increased to 36%.^[^ ^2^, ^3^ ^]^ Elevated blood ANP and BNP levels are common biomarkers for late‐onset cardiotoxicity, significantly higher than cTnT and cTnI levels.^[^ ^4^ ^]^ Despite doxorubicin dosing guidelines,^[^ ^5^ ^]^ lack of robust predictive biomarkers prohibits the prevention of DIC. Pre‐existing cardiovascular disease provides some guidelines but due to the late onset nature, DIC remains a risk for cancer patients.

Prior to human induced pluripotent stem cell derived cardiomyocytes technology (hiPSC‐CMs),^[^ ^6^, ^7^ ^]^ human cardiomyocytes are hard to obtain and cannot be cultured to study disease mechanism.^[^ ^8^ ^]^ Using hiPSC‐CMs derived from Dox‐treated patients that developed DIC or not, we showed that doxorubicin sensitive hiPSC‐CMs develop mitochondrial dysfunction and apoptosis upon doxorubicin challenge.^[^ ^9^ ^]^ More recently, we demonstrated that exogenous mitochondrial transplantation can *dilute* doxorubicin toxicity in both hiPSC‐CMs and mice.^[^ ^10^ ^]^ Whether mitochondrial amount determines DIC toxicity remains elusive.

The leakage of mitochondrial contents requires passage through the mitochondrial permeability transition pore (mPTP).^[^ ^11^, ^12^ ^]^ Two pro‐apoptotic members of the BCL‐2 family, BAX and BAK, are involved in inducing MOMP that drives the release of cytochrome C and other soluble factors, including mtDNA, into the cytoplasm.^[^ ^13^ ^]^ Evidence show that cytoplasmic mtDNA is recognized by DNA sensor cGAS, and cGAS‐STING pathway activation can drive inflammation and cellular senescence.^[^ ^14^ ^]^ The leakage of double‐stranded DNA into the cytoplasm activates the cellular cGAS‐STING pathway, which primarily triggers an intracellular autoimmune response. Cytoplasmic DNA, as a significant danger signal, is recognized by cGAS, promoting downstream activation of STING and consequently causing an inflammatory storm in the cell, accelerating cellular senescence. cGAS recognizes and is activated by DNA ligands, assembling into dimers. Active cGAS produces cGAMP, which binds to the stimulator of interferon genes (STING), leading to TANK‐binding kinase 1 (TBK1)‐dependent phosphorylation of interferon regulatory factor 3 (IRF3). The active IRF3 dimer translocate to the nucleus, activating the transcription of type I interferon genes and inducing the expression of numerous inflammatory factors.^[^ ^14^ ^]^

Here, we demonstrate DIC toxicity is dependent on mitochondrial amount, and molecularly, mtDNA leakage drives cGAS‐STING dependent inflammation and myocardial senescence. In cancer patient peripheral blood mononuclear cells (PBMCs), we find a negative correlation between mitochondrial copy number and heart failure biomarkers after anthracycline chemotherapy. Using hiPSC‐CM model we generated conditions with different mitochondrial amounts and demonstrate that lower mitochondrial amount correlated with stronger DIC. Molecularly, we show that doxorubicin treatment induced mitochondrial membrane leakage (through BAX, BAK, VDAC1, and VDAC3 channels), increased cytosolic mtDNA level activated mtDNA‐cGAS‐STING inflammatory pathway, and resulted myocardial senescence. Through pharmacological, cardiac‐specific knock‐out of STING, and over‐expression of mitochondrial localized DNase1 blocks doxorubicin induced mtDNA‐cGAS‐STING activation and ameliorates DIC toxicity.

## 2 Results

### 2.1 Patients with a Higher Mitochondrial Copy Number may have a Better Prognosis in Anthracycline‐Based Chemotherapy

**Figure 1:**
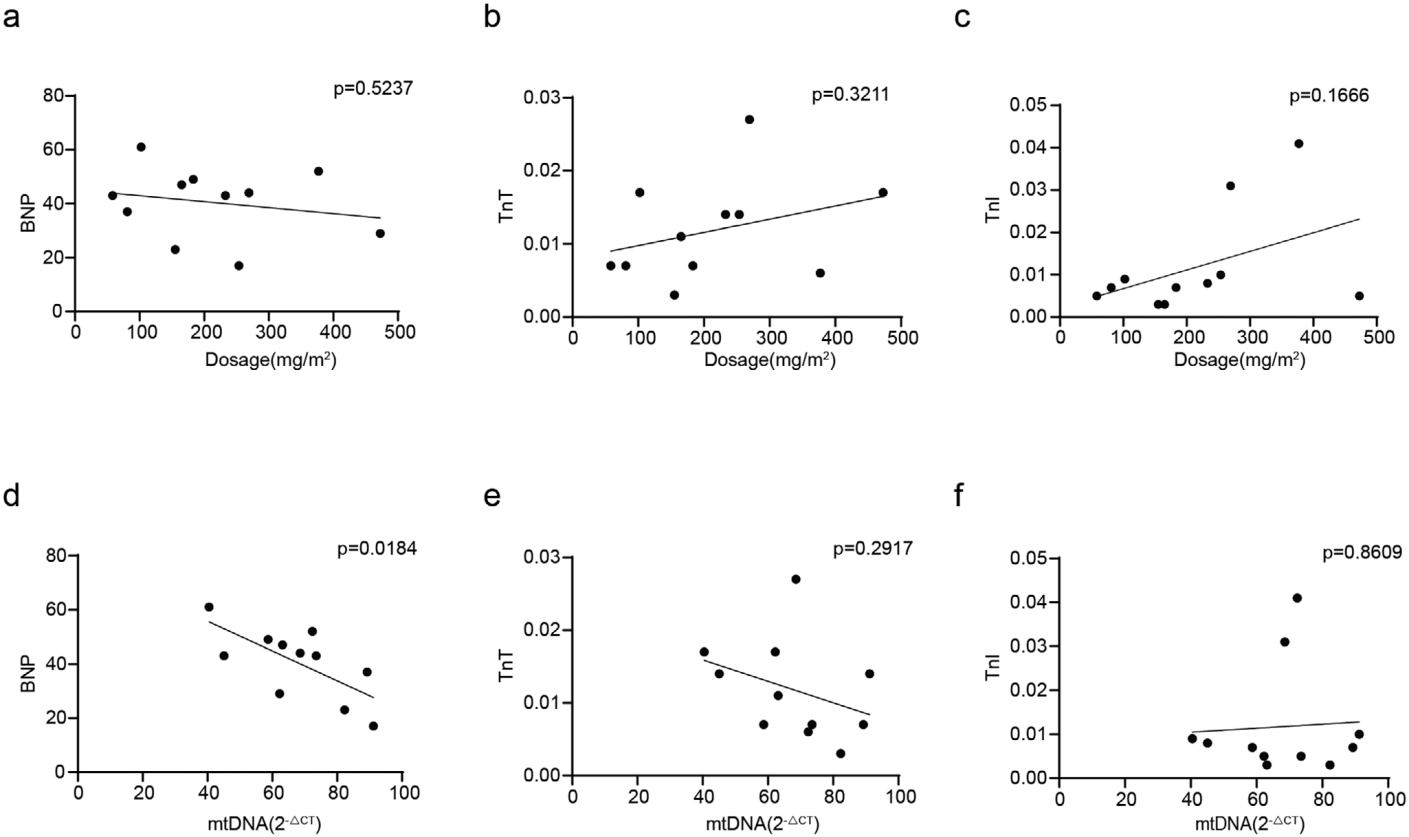
Patients with a higher mitochondrial copy number may have a better prognosis in anthracycline‐based chemotherapy. a) Correlation between doxorubicin dosage and plasma BNP levels in patients (*n* = 11). b) Correlation between doxorubicin dosage and plasma cTnT levels in patients (*n* = 11). c) Correlation between doxorubicin dosage and plasma cTnI levels in patients (*n* = 11). d) Correlation between mitochondrial DNA copy number and plasma BNP levels (*n* = 11). e) Correlation between mitochondrial DNA copy number and plasma TnT levels (*n* = 11). f) Correlation between mitochondrial DNA copy number and plasma TnI levels (*n* = 11).

Previously we observed that hiPSC‐derived cardiomyocytes from doxorubicin sensitive patients exhibit lower mitochondrial amount compared to hiPSC‐CMs from patients that did not develop DIC.^[^ ^9^ ^]^ To investigate whether lower mitochondrial levels correlated to severe DIC toxicity, with patients written consent, we recruited cancer patients (*n* = 11) and collected 8 mL of whole blood prior to their anthracycline chemotherapy and monitored their heart failure biomarkers (BNP [early heart failure], cTnT and cTnI [late heart failure]). DNA was extracted from PBMCs and mtDNA copy number was measured using RT‐qPCR as previously described.^[^ ^15^ ^]^ In our cohort, we found no correlation between chemotherapy dosage, BNP, and cTnI (**Figure** **1**a‐c). However, BNP levels correlated negatively with mitochondrial copy number suggesting early cardiac damage was more prevalent in patients with lower mitochondrial content (Figure 1d). Additionally, we observed that changes in cTn levels were not significantly correlated with either the mtDNA copy number in PBMCs or the drug dosage (Figure 1e,f), which is consistent with previous findings that change in cTn levels is a late onset heart failure biomarker for DIC.^[^ ^5^ ^]^ These results support a model by which DIC is correlated with mitochondrial levels.

### 2.2 Cardiomyocytes with Different Mitochondrial Copy Numbers Exhibit Varying Tolerance to Doxorubicin

**Figure 2:**
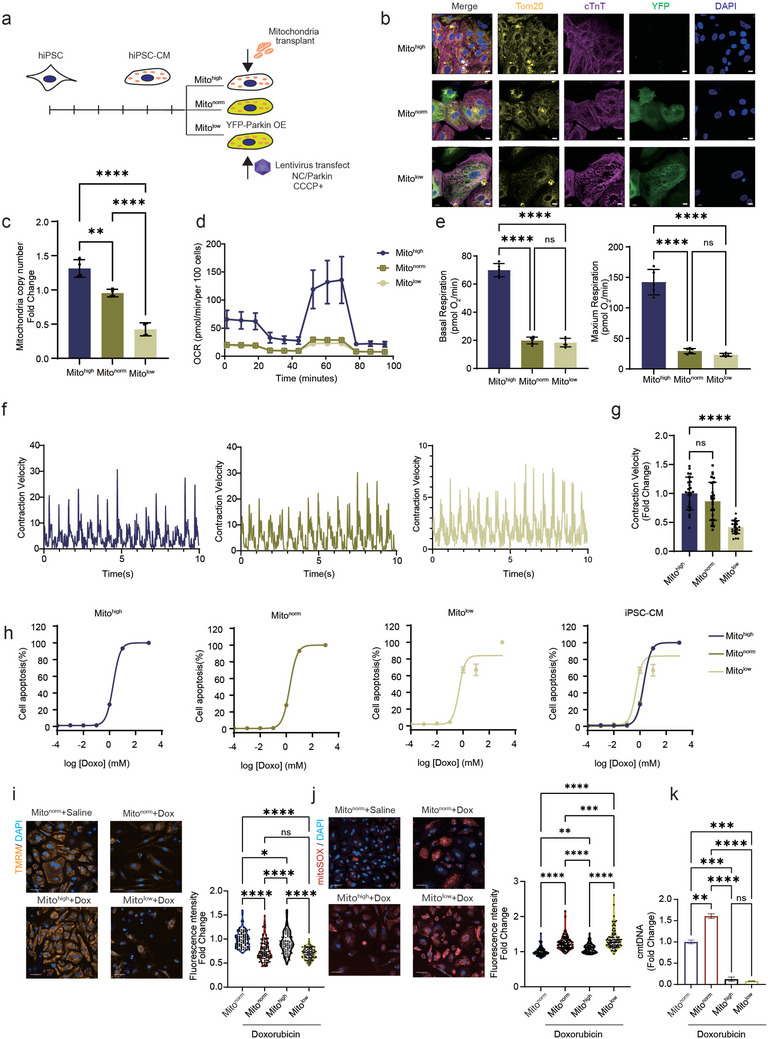
HiPSC‐CMs with varying mitochondrial DNA copy numbers exhibit different levels of doxorubicin tolerance. a) Representation of cells with varying mitochondrial DNA copy numbers. b) Confocal microscopy shows hiPSC‐CMs with three distinct mitochondrial DNA copy numbers, TOM20 (yellow), cTnT (purple), YFP (green), nuclear (blue), scale bar 10 µm. c) qPCR showing differences in mitochondrial DNA copy numbers among three distinct hiPSC‐CMs types, *n* = 3, quantification values are expressed as mean ± SEM. d) Seahorse analysis showing differences in basal oxygen consumption among hiPSC‐CMs with three distinct mitochondrial DNA copy numbers, *n* = 6, quantification values are expressed as mean ± SEM. e) hiPSC‐CMs with three distinct mitochondrial DNA copy numbers show significant differences in maximal oxygen consumption and basal respiratory oxygen consumption, *n* = 6, quantification values are expressed as mean ± SEM. f,g) hiPSC‐CMs with higher mitochondrial DNA copy numbers exhibit stronger beating capacity, *n* = 30, quantification values are expressed as mean ± SEM. h) Apoptosis curves show that hiPSC‐CMs with fewer mitochondrial DNA copy numbers have lower doxorubicin tolerance, *n* = 3, quantification values are expressed as mean ± SEM, TUNEL assay after doxorubicin treated 24 h. i) TMRM staining shows that after doxorubicin treatment, hiPSC‐CMs with more mitochondria maintain mitochondrial membrane potential (orange), whereas hiPSC‐CMs with fewer mitochondria exhibit decreased membrane potential, doxorubicin 1 µM, 24 h, scale bar 100 µm, *n* = 30, quantification values are expressed as mean ± SEM. j) mito‐SOX staining shows that after doxorubicin treatment, hiPSC‐CMs with more mitochondria produce less mitochondrial reactive oxygen species (superoxide, red), while hiPSC‐CMs with fewer mitochondria produce more mitochondrial superoxide, doxorubicin 1 µM, 24 h, scale bar 100 µm, *n* = 30, quantification values are expressed as mean ± SEM. k) RT‐qPCR analysis revealed that following doxorubicin treatment 1 µM, 24 h, mito^high^ cells exhibited reduced cytosolic mtDNA leakage, *n* = 2, quantification values are expressed as mean ± SEM.

To test if varying mitochondrial amounts alters DIC sensitivity, we generated hiPSC‐CMs with high (mito^high^), normal (mito^norm^) and low (mito^low^) mitochondrial levels and was confirmed by TOM20 immunofluorescence staining (**Figure** **2**a,b). cTnT levels and structure showed no differences between mito^high^, mito^norm^ and mito^low^ hiPSC‐CMs (Figure 2b). For mito^high^ condition, we transplanted purified HL‐1 mitochondria into hiPSC‐CMs using previously established protocol,^[^ ^10^ ^]^ and successfully increased mitochondrial DNA copy number by 50% (Figure 2c). For mito^low^ condition, we used the YFP‐Parkin add CCCP system developed by Passos’ group,^[^ ^16^ ^]^ and generated hiPSC‐CMs with 50% reduction of mitochondrial copy number (Figure 2c). Functionally, a decrease in mitochondrial amount (mito^low^) resulted in a decrease in mitochondrial respiration capacity (Figure 2d,e) and myocardial contractility (Figure 2f,g); excess mitochondrial amount (mito^high^) boosted mitochondrial respiration capacity but did not alter myocardial contractility (Figure 2f,g). In parallel, mito^high^ mito^norm^ mito^low^ mouse‐derived cardiomyocyte cell line HL‐1 and cervical cancer cell line HeLa were also constructed (Figure S1a‐c, Supporting Information).

To confirm that mitochondrial transplantation and clearance do not introduce additional apoptotic responses, we performed TUNEL staining in mito^high^, mito^norm^, and mito^low^ hiPSC‐CMs. The results showed that inducing mitochondrial clearance did not trigger apoptosis in these cells (Figure S1d, Supporting Information), demonstrating that this system is suitable for subsequent assessments of doxorubicin‐induced apoptotic toxicity. To determine if DIC toxicity is inversely correlated with mitochondrial amount, we then challenged mito^high^, mito^norm^, and mito^low^ hiPSC‐CMs with varying concentration of doxorubicin and used apoptosis as a readout of DIC. Compared to mito^norm^, mito^low^ hiPSC‐CMs apoptosis curve shifted leftward demonstrating increased toxicity, no difference was observed between mito^high^ and mito^norm^ hiPSC‐CMs (Figure 2h; Figure S1e, Supporting Information). Compared to mito^norm^ hiPSC‐CMs, mito^high^ hiPSC‐CMs retained higher mitochondrial membrane potential (Figure 2i; Figure S1f, g, Supporting Information) and lower mitochondrial superoxide after 1µM doxorubicin 24 h challenge (Figure 2j; Figure S1f, g, Supporting Information). Mito^low^ hiPSC‐CMs exhibited lower mitochondrial membrane potential (Figure 2i; Figure S1f, g, Supporting Information) and higher mitochondrial superoxide (Figure 2j; Figure S1f,g, Supporting Information) compared to mito^norm^ hiPSC‐CMs. To confirm that doxorubicin induces mitochondrial damage and triggers the release of mitochondrial contents, cytosolic mtDNA was extracted from mito^high^, mito^low^, and mito^norm^ cells following doxorubicin treatment. The mtDNA copy number in the cytosol was quantified using RT‐qPCR. The mito^high^ cells subjected to mitochondrial transplantation exhibited reduced mtDNA leakage following doxorubicin treatment, demonstrating that mitochondrial transplantation as an increased mitochondrial copy number can alleviate doxorubicin‐induced mtDNA leakage (Figure 2k). However, mito^low^ cells also showed reduced mtDNA leakage after doxorubicin treatment, potentially due to their intrinsically lower baseline levels of mitochondria and mtDNA. These results suggest that hiPSC‐CMs with more mitochondrial amounts are more resistant to doxorubicin induced toxicity.

### 2.3 Doxorubicin Activates mtDNA‐cGAS‐STING through BAX/BAK and VDAC Channels

**Figure 3:**
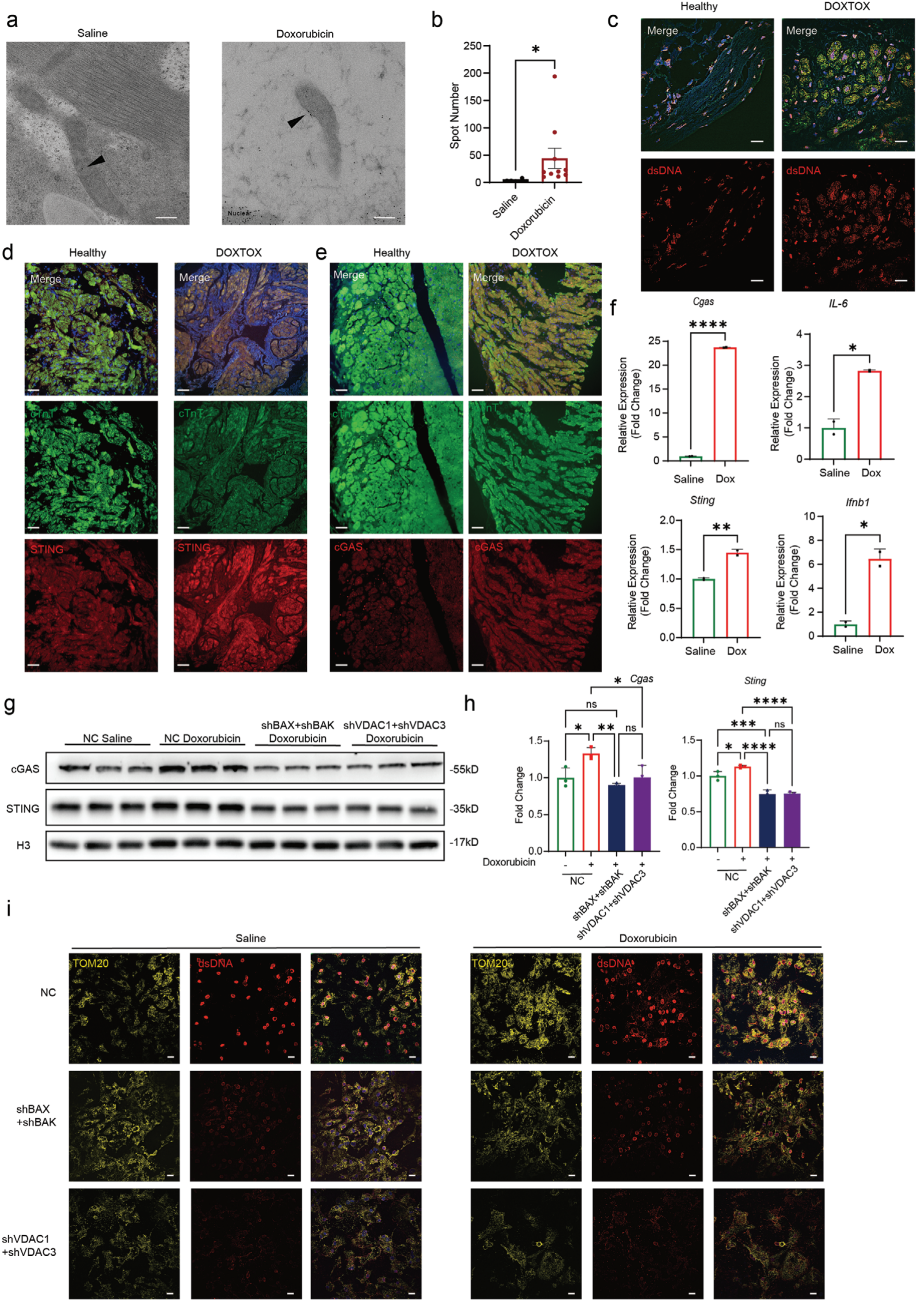
Doxorubicin induces mitochondrial DNA release into the cytoplasm through BAX/BAK and VDAC channels, activating the cGAS‐STING pathway. a,b) Immunoelectron microscopy shows mitochondrial leakage into the cytoplasm in hiPSC‐CMs treated with doxorubicin. Gold particles indicated by arrows represent mtDNA, doxorubicin 1 µM, 24 h, scale bar = 200 nm, *n* = 12, quantification values are expressed as mean ± SEM. c) Immunofluorescence shows localization of dsDNA (red) and mitochondria (TOM20, green) in heart section slide, scale bar = 20µm. d) Immunofluorescence shows expression of STING (red) and cTnT (green) in heart section slide, scale bar = 50 µm. e) Immunofluorescence shows expression of cGAS (red) and cTnT (green) in heart section slide, scale bar = 50 µm. f) qPCR shows activation of the cGAS‐STING pathway and increased expression of downstream inflammatory cytokines in hiPSC‐CMs after doxorubicin treatment, doxorubicin 1 µM, 24 h, *n* = 2, quantification values are expressed as mean ± SEM. g,h) Immunoblotting demonstrates that knock down of BAX/BAK and VDAC prevents doxorubicin‐induced activation of the cGAS‐STING pathway in hiPSC‐CMs, doxorubicin 1 µM, 24 h, *n* = 3, quantification values are expressed as mean ± SEM. i) Immunofluorescence shows localization of dsDNA (red) and mitochondria (TOM 20, yellow) in hiPSC‐CMs, scale bar = 20 µm.

Given that mPTP opening can cause a drop in mitochondrial membrane potential,^[^ ^17^ ^]^ we hypothesize that doxorubicin can induce mPTP opening leading to mtDNA leakage. To test this, we used gold particles labeled dsDNA antibodies and examined presence of mitochondrial DNA in doxorubicin treated hiPSC‐CMs by transmission electron microscopy (TEM) as previously described.^[^ ^18^, ^19^ ^]^ In saline‐treated hiPSC‐CMs, mitochondria and sarcomere structures were intact and dsDNA signals remain localized within the nucleus and mitochondria (**Figure** **3**a). However, in doxorubicin‐treated hiPSC‐CMs, dsDNA signals were present on ruptured mitochondrial outer membrane and accumulated in the cytoplasm (Figure 3a,b). To confirm this observation clinically, we stained both health and doxorubicin treated patient heart sections for dsDNA and mitochondria (TOM20) co‐localization (Figure 3c). Compared to healthy control, cardiac sections from doxorubicin‐treated patients exhibited significantly elevated levels of cytoplasmic dsDNA (Figure 3c). MtDNA leakage after doxorubicin induction was also observed in HL‐1 cells by immunofluorescence (Figure S2a, Supporting Information). Moreover, doxorubicin treated HL‐1 cells exhibit higher levels of cytosolic mtDNA (cmtDNA) relative to non‐treated HL‐1 cells (Figure S2b, Supporting Information). These observations suggest mitochondrial ultrastructure loss due to doxorubicin challenge leads to mitochondrial DNA leakage.

It has been suggested that cytosolic mtDNA induces cGAS‐STING pathway and leads to inflammatory response.^[^ ^14^ ^]^ To investigate whether doxorubicin treatment activates the cGAS‐STING pathway in cardiomyocytes, we performed immunofluorescence staining on patient cardiac sections for cGAS and STING. Compared to healthy control, the expression levels of cGAS and STING were significantly higher in DIC sample (Figure 3d,e). In agreement, we observed an increase in cGAS and STING protein levels and downstream activation in doxorubicin treated hiPSC‐CMs (Figure 3f; Figure S2d, Supporting Information). These results suggest that doxorubicin can induce mtDNA‐cGAS‐STING pathway activation.

Previously it has been shown that mtDNA can leak out through BAX,^[^ ^19^ ^]^ BAK, VDAC1, or VDAC3^[^ ^18^ ^]^ channels. To test which mPTP channel is activated by doxorubicin challenge, we first analyzed cardiomyocytes RNA‐seq data generated from doxorubicin and vehicle treated animals. Compared to vehicle, cardiomyocytes from doxorubicin animals showed an increase in *Bax*, *Bak*, *Vdac1*, *and Vdac3* gene expression (Figure S2e, Supporting Information). To verify whether mtDNA is leaked through one of these mPTP channels, we constructed shRNAs targeting BAX, BAK, VDAC1, and VDAC3 and infected hiPSC‐CMs. The shRNA is capable of knocking down both *Bax* and *Bak*, or *Vdac1*, and *Vdac3* (Figure S2f, Supporting Information). We found that knocking down BAX, BAK, VDAC1, and VDAC3 decreased cGAS and STING protein levels (Figure 3g,h) and reduced cytoplasmic mtDNA (Figure 3i, dsDNA staining) after doxorubicin treatment. Further, pretreating HL‐1 cells with inhibitors of BAI1 (BAX, BAK), and VBIT‐4 (VDAC) before doxorubicin challenge prevented cGAS‐STING pathway activation (Figure S2c, Supporting Information). These results demonstrate that doxorubicin drives myocardial mPTP opening and results in cGAS‐STING pathway activation.

### 2.4 mtDNA Leakage‐Activated cGAS‐STING Pathway Promotes Senescence in Myocardial Cells

**Figure 4:**
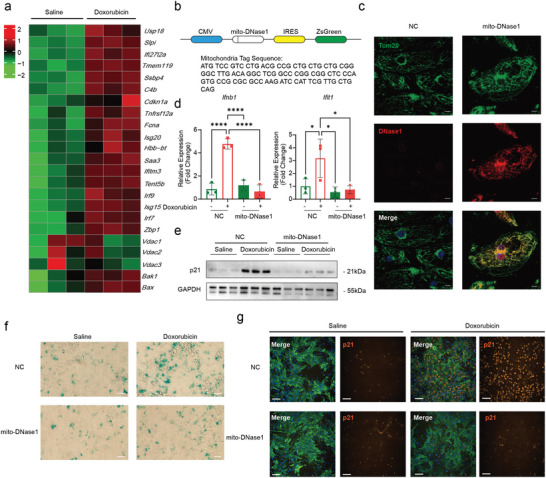
MtDNA leakage‐activated cGAS‐STING pathway promotes senescence in cardiomyocytes. a) Bulk RNA‐seq reveals increased expression of inflammatory cytokines in myocardial cells after doxorubicin treatment mice, *n* = 3. b) A mitochondria‐targeted DNase1 has been designed to eliminate extruded mtDNA post‐doxorubicin treatment. c) Confocal microscopy shows localization of DNase1 (red) on mitochondria (TOM20, green) in infected hiPSC‐CMs, scale bar = 10 µm. d) qPCR reveals a significant decrease in inflammatory cytokine levels in hiPSC‐CMs expressing mitochondria‐targeted DNase1 following doxorubicin treatment, *n* = 3, quantification values are expressed as mean ± SEM. e) Immunoblotting demonstrate a significant decrease in p21 levels in hiPSC‐CMs expressing mitochondria‐targeted DNase1 following doxorubicin treatment, doxorubicin 1 µM, 24 h, *n* = 3, quantification values are expressed as mean ± SEM. f) SA‐β‐gal staining shows a significant reduction in the proportion of senescence cells in hiPSC‐CMs expressing mitochondria‐targeted DNase1 following doxorubicin treatment, scale bar = 40 µm. g) p21 immunofluorescence staining shows a significant reduction in the proportion of p21 positive cells in neonatal mouse ventricular myocytes expressing mitochondria‐targeted DNase1 following doxorubicin treatment, scale bar = 100 µm.

We and others have shown that doxorubicin can activate the p53 pathway^[^ ^20^ ^]^ and drive cellular senescence^[^ ^21^ ^]^ in a mitochondria‐dependent manner,^[^ ^22^ ^]^ however, whether p53 occur downstream of cGAS‐STING pathway remains unknown. To confirm abovementioned observations, we re‐analyzed our RNA‐seq data and found that compared to control mice, doxorubicin‐treated mouse cardiomyocytes exhibited high expression levels of cGAS‐STING downstream inflammatory factors (**Figure** **4**a). To inhibit doxorubicin‐induced mtDNA leakage, we constructed a DNase1 fused with a mitochondrial targeting peptide (mito‐DNase1) and over expressed in hiPSC‐CMs (Figure 4b). Immunofluorescence staining confirmed colocalization of DNase1 and TOM 20 (Figure 4c). Compared to control hiPSC‐CMs, doxorubicin treatment did not induce the expression of inflammatory factors in mito‐DNase1 overexpressed hiPSC‐CMs (Figure 4d), preventing mtDNA‐cGAS‐STING activation. Further, mito‐DNase1 over expression prevented myocardial senescence marked by p53, p21, p16 (Figure 4e,g; Figure S2g‐i, Supporting Information), and SA‐β‐Gal staining (Figure 4f). These results demonstrate that doxorubicin induced myocardial senescence is driven by mtDNA leakage and over expression of mito‐DNase1 halts cGAS‐STING activation.

### 2.5 Heart Mitochondrial Transplantation Alleviates Doxorubicin‐Induced Cardiotoxicity

**Figure 5:**
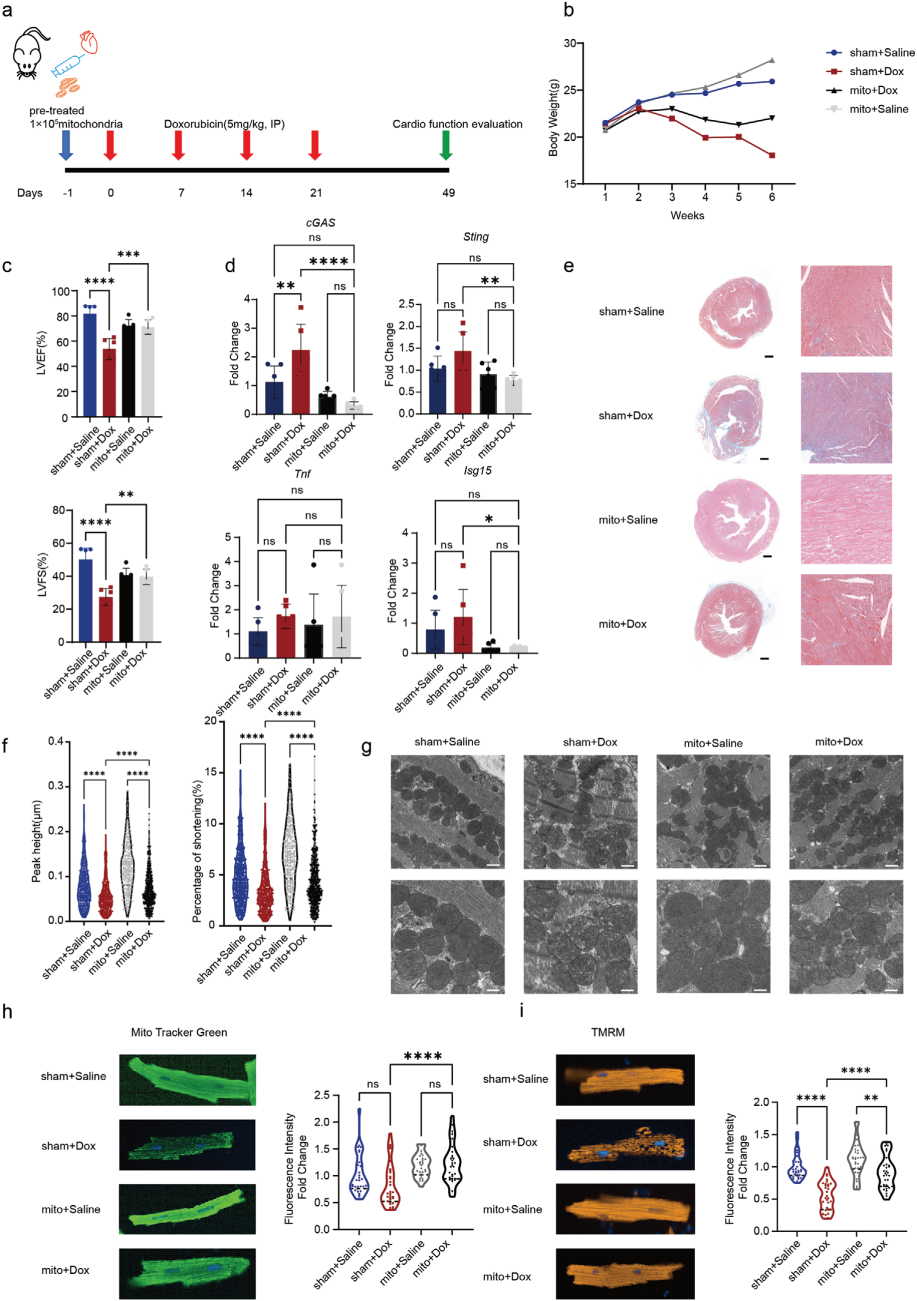
Heart mitochondrial transplantation alleviates doxorubicin‐induced cardiotoxicity. a) Schematic diagram of the chronic doxorubicin‐induced heart failure model with in situ mitochondrial transplantation. b) Mouse weight tracking shows that in situ transplantation of cardiac mitochondria protects against weight loss following doxorubicin treatment, *n* = 5, quantification values are expressed as mean ± SEM. c) Echocardiography shows that in situ transplantation of cardiac mitochondria partially alleviates doxorubicin‐induced cardiac dysfunction, *n* = 5, quantification values are expressed as mean ± SEM. d) RT‐qPCR demonstrate a significant decrease in cGAS and STING levels and downstream inflammation factors in AMCM cells of in situ mitochondria transplant mouse following doxorubicin treatment, *n* = 3, quantification values are expressed as mean ± SEM. e) Masson staining shows that in situ transplantation of cardiac mitochondria partially alleviates doxorubicin‐induced cardiac fibrosis, with blue indicating fibrotic areas in mouse heart section, scale bar = 200 µm. f) Ionoptix detection of isolated adult mouse cardiomyocytes under simulated physiological beating conditions shows that in situ transplantation of cardiac mitochondria alleviates doxorubicin‐induced cardiotoxicity, *n* = 100, quantification values are expressed as mean ± SEM. g) Transmission electron microscopy shows that in situ transplantation of cardiac mitochondria alleviates doxorubicin‐induced mitochondrial rupture, membrane swelling, and cristae fragmentation, scale bar = 500 nm. h) MitoTracker Green staining of isolated adult mouse cardiomyocytes shows that in situ transplantation of cardiac mitochondria increases mitochondrial copy numbers reduced by doxorubicin, *n* = 100, quantification values are expressed as mean ± SEM. i) TMRM staining of isolated adult mouse cardiomyocytes shows that in situ transplantation of cardiac mitochondria partially alleviates doxorubicin‐induced mitochondrial membrane potential decline, *n* = 100, quantification values are expressed as mean ± SEM.

Previously we have demonstrated that mitochondrial transplantation into cardiomyocytes prevents doxorubicin induced apoptosis,^[^ ^10^ ^]^ however, we do not know if an increase in mitochondrial amount prevents doxorubicin induced mtDNA leakage. We performed single dose intramyocardial mitochondrial transplantation before doxorubicin challenge in 8‐week‐old C57/Bl6J male mice, as previously described^[^ ^10^, ^23^ ^]^ (**Figure** **5**a). To investigate the retention duration of mitochondria in the mouse heart, we isolated mitochondria from human hiPSC‐derived cardiomyocytes and labeled them with the fluorescent dye DilC. A total of 10⁵ labeled mitochondria were injected intramyocardially into the apex of mouse hearts. Heart tissues were collected at 4 h, 24 h, 48 h, 72 h, and 1 week post‐injection. Fluorescent mitochondria were visualized in frozen cardiac sections at all time points and injected mitochondria persisted in the mouse heart for at least one week (Figure S3a, Supporting Information). Additionally, RT‐qPCR was performed to assess mouse mtDNA copy numbers in mouse hearts following mitochondrial transplantation, we observed an increase in mouse mtDNA copy number post‐transplantation 4 h, 1 week, and 6 weeks (Figure S3b, Supporting Information). This suggests that mitochondrial transplantation may stimulate endogenous mitochondrial biogenesis in the mouse heart, thereby increasing mitochondrial copy numbers. Mitochondrial transplanted animals exhibited delayed doxorubicin induced body weight loss (Figure 5b). Echocardiography shows mitochondrial transplanted mice exhibit nearly no change in LVEF and LVFS after doxorubicin stimulation (Figure 5c; Figure S3f, Supporting Information). Mitochondrial transplantation did prevent mtDNA leakage induced cGAS‐STING (Figure 5d) activation and myocardial senescence. Histologically, Masson's staining shows that mitochondrial transplanted mic exhibited significantly reduced fibrosis levels after doxorubicin stimulation (Figure 5e). Functionally, purified adult mouse cardiomyocytes (AMCMs) showed that the cardiomyocyte contraction function in mice that underwent mitochondrial transplantation was significantly better than that in AMCMs from mice that did not undergo mitochondrial transplantation after doxorubicin induction (Figure 5f; Figure S3d, Supporting Information). Following doxorubicin treatment, mitochondrial status improved with mitochondrial transplantation. Mitochondrial copy number in pre‐mitochondrial transplant cardiomyocytes did not decrease after doxorubicin damage (Figure S3c, Supporting Information). Additionally, mitochondrial morphology and structure in heart sections of mice treated with doxorubicin were protected following mitochondrial transplantation (Figure 5g). MitoTracker Green staining showed that mitochondrial copy number was preserved in AMCMs from mice that received mitochondrial transplantation before doxorubicin treatment (Figure 5h). JC‐1 staining revealed that mitochondrial membrane potential was also preserved under the same conditions (Figure 5i). RNA‐seq analysis of purified AMCMs demonstrated that numerous genes upregulated by doxorubicin induction were reversed by mitochondrial transplantation (Figure S3e, Supporting Information). These results indicate that preemptively increasing mitochondrial copy number through mitochondrial transplantation can mitigate acute DIC (Figure 2h) but is unable in preventing the cardiac senescence.

### 2.6 Myocardial Specific *Sting* Knockout or Over Expression of Mito‐DNase1 both Protect DIC

**Figure 6:**
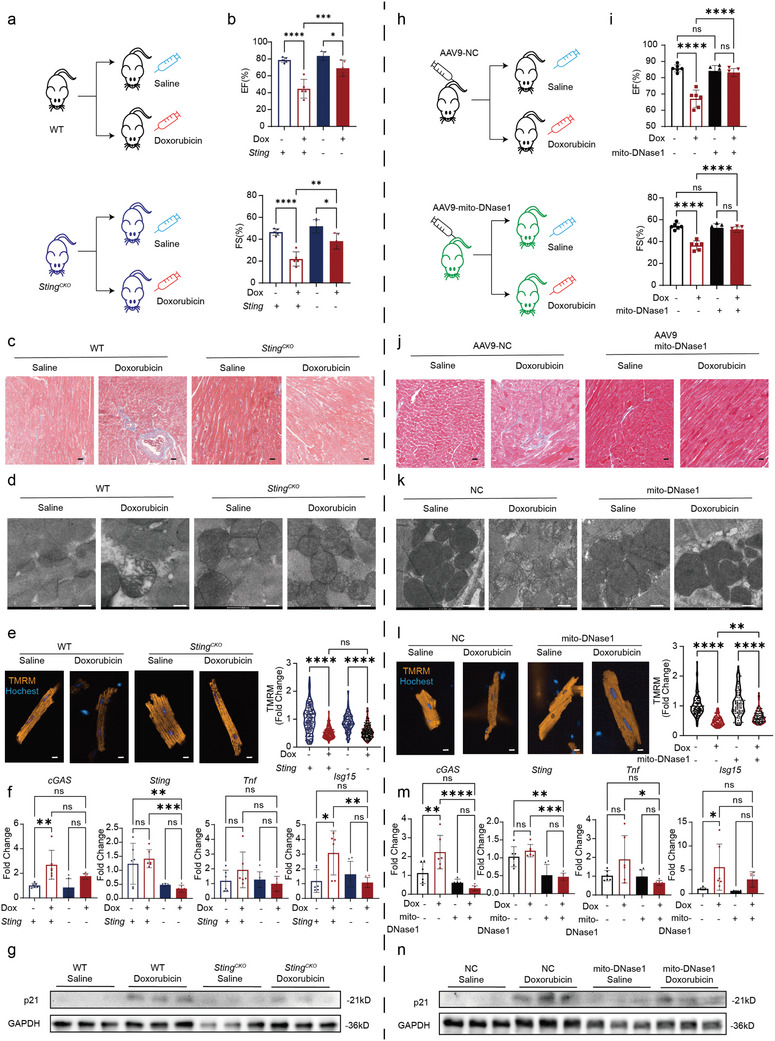
Myocardial specific STING knockout and overexpression of mitochondria‐targeted DNase1 both confer protection against doxorubicin‐induced cardiotoxicity. a,h) Schematic representation of the chronic heart failure model induced by doxorubicin in myocardial specific STING knockout and mitochondria‐targeted DNase1 OE mice. b,i) Echocardiography shows that myocardial specific STING knockout and mitochondria‐targeted DNase1 OE partially alleviates doxorubicin‐induced cardiac dysfunction, *n* = 5, quantification values are expressed as mean ± SEM. c,j) Masson staining shows that myocardial specific STING knockout and mitochondria‐targeted DNase1 OE partially alleviates doxorubicin‐induced cardiac fibrosis, blue indicating fibrotic areas, scale bar = 200 µm. d,k) Transmission electron microscopy shows that myocardial specific STING knockout and mitochondria‐targeted DNase1 OE partially alleviates doxorubicin‐induced mitochondrial rupture, membrane swelling, and cristae fragmentation, scale bar = 500 nm. e,l) TMRM staining of isolated adult mouse cardiomyocytes shows that STING knockout and mitochondria‐targeted DNase1 OE partially alleviates doxorubicin‐induced mitochondrial membrane potential decline, *n* = 100, quantification values are expressed as mean ± SEM. f,m) RT‐qPCR demonstrate a significant decrease in cGAS and STING levels and downstream inflammation factors in AMCMs of STING knockout and mitochondria‐targeted DNase1 OE mice following doxorubicin treatment, *n* = 3, quantification values are expressed as mean ± SEM. g,n) Immunoblotting shows that myocardial specific STING knockout and mitochondria‐targeted DNase1 OE partially reduces doxorubicin‐induced p21 expression, *n* = 3.

**Figure 7:**
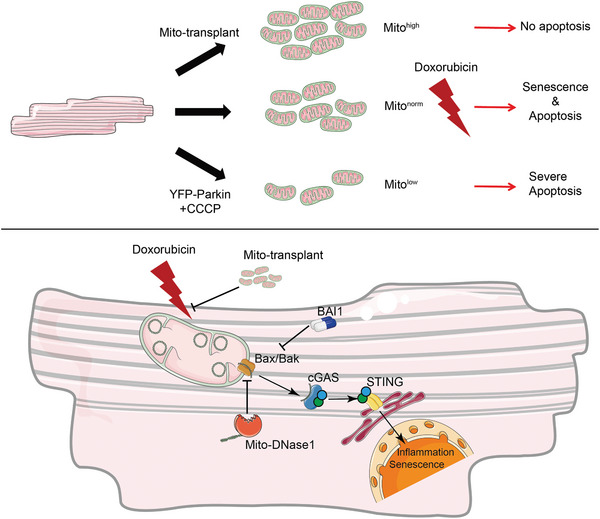
Schematic presentation showing the signaling mechanism and function of the mtDNA‐cGAS‐STING pathway in doxorubicin injury and rescue strategies.

To test if inhibition of leakage mtDNA and cGAS‐STING pathway will prevent the onset of DIC in vivo, we either challenged *Sting^CKO^* animals with doxorubicin (Dox) (**Figure** **6**a) or overexpressed (OE) mito‐DNase1 (AAV9‐cTnT‐mito‐DNase1) prior to Dox challenge (Figure 6h). Functionally, *Sting^CKO^* (Figure 6b; Figure S4a, Supporting Information) or mito‐DNase1 OE (Figure 6i; Figure S4h, Supporting Information) prevented the onset of cardiac systolic dysfunction upon Dox challenge measured by LVEF and LVFS. Histologically, Masson's staining shows that both *Sting^CKO^* (Figure 6c) and mito‐DNase1 OE mice (Figure 6j) exhibited significantly reduced fibrosis levels after doxorubicin stimulation. Further, *Sting^CKO^* (Figure 6d) or mito‐DNase1 OE (Figure 6k) adult mouse heart section shows healthier mitochondria under TEM analysis, and shows higher mitochondrial membrane potential after doxorubicin stimulation (Figure 6e,l). RT‐qPCR (reversed transcription quantitative polymerase chain reaction) of AMCMs shows that cGAS‐ STING expression levels and downstream inflammation factors were also reduced in *Sting^CKO^* (Figure 6f) and mito‐DNase1 (Figure 6m) OE mice after doxorubicin stimulation. Immunoblotting demonstrated that the expression level of p21 was also reduced in *Sting^CKO^* (Figure 6g; Figure S4g, Supporting Information) and mito‐DNase1 OE (Figure 6n; Figure S4n, Supporting Information) mice after doxorubicin stimulation. Furthermore, *Sting^CKO^* and mito‐DNase1 OE AMCMs showed significantly better contractile functions in Dox‐challenged *Sting^CKO^* (Figure S4‐f, Supporting Information) and mito‐DNase1 OE AMCMs compared to control AMCMs. These results suggest that both *Sting^CKO^* and mito‐DNase1 OE can partially rescue doxorubicin induced cardiotoxicity and myocardial senescence. Collectively, these data demonstrate that upstream inactivation of cGAS‐STING pathway (mito‐DNase1 and *Sting^CKO^*) confers cardio‐protection against DIC compared to mitochondrial transplantation (**Figure** **7**).

## 3 Discussion

In this study, we demonstrate that mitochondrial content determines myocardial susceptibility to doxorubicin. Molecularly, we show that doxorubicin disrupts mitochondrial membrane integrity by opening mitochondrial permeability transition pore BAX, BAK, and VDAC, leading to mtDNA leakage into the cytoplasm. Subsequent activation of cGAS‐STING pathway drives myocardial senescence. Genetic ablation of myocardial STING or degradation of cytosolic mtDNA by overexpression of mito‐DNase1 or mitochondrial transplantation halts cGAS‐STING activation and rescues cardiac dysfunction (Figure 7).

Doxorubicin‐induced cardiotoxicity is often late onset in cancer survivors that were treated for childhood cancer.^[^ ^24^ ^]^ Doxorubicin‐induced cardiotoxicity mechanisms include oxidative stress,^[^ ^25^ ^]^ inflammation,^[^ ^26^, ^27^ ^]^ mitochondrial damage,^[^ ^28^, ^29^ ^]^ calcium homeostasis imbalance,^[^ ^30^, ^31^ ^]^ autophagy,^[^ ^32^, ^33^ ^]^ apoptosis,^[^ ^34^, ^35^ ^]^ and ferroptosis.^[^ ^20^, ^36^, ^37^ ^]^ However, what pre‐disposition makes an individual susceptible to DIC remains debated given that cumulative dosage cannot. Currently, there are no reports on the use of PBMC responses to predict doxorubicin‐induced cardiotoxicity. We observed a negative correlation between changes in blood BNP levels following doxorubicin treatment and mitochondrial copy numbers in pre‐treatment PBMCs from patients. However, as mitochondrial copy numbers in PBMCs do not directly reflect those in cardiomyocytes and it is impractical to obtain pre‐treatment cardiac tissue samples from patients, we developed hiPSC‐derived cardiomyocyte models with varying mitochondrial copy numbers to address this limitation. Using patient hiPSC‐CMs, it was first demonstrated that DIC sensitivity in patients can be recapitulated in patients’ hiPSC‐CMs ^[^ ^38^ ^]^ and this pre‐disposition can be partly explained by SNPs on *RARG* and *SLC28A3*.^[^ ^39^, ^40^ ^]^ Previously, we have demonstrated that mitochondrial transplantation can *dilute* and prevent doxorubicin‐induced myocardial apoptosis while metabolic compliant mitochondria can restore contractile function after doxorubicin challenge.^[^ ^10^ ^]^ With results shown in this study, we provide an alternative explanation for DIC pre‐disposition. Currently, no studies have directly investigated the relationship between doxorubicin‐induced cardiomyopathy and mitochondrial copy number. In keep with the original hiPSC‐CM study,^[^ ^9^ ^]^ where mitochondrial amount setpoint is maintained during reprogramming (fibroblast to hiPSC) and differentiation (hiPSC to hiPSC‐CMs), here we show that mito^low^ hiPSC‐CMs exhibit greater DIC susceptibility compared to mito^norm^ and mito^high^ hiPSC‐CMs. This cardio‐protection also confirmed in vivo where animals were pre‐transplanted with mitochondria prior to Doxorubicin challenge.

The cGAS‐STING pathway is a classic autoimmunity pathway that is activate in presence of cytosolic DNA. Aberrant cGAS‐STING activation has been observed in cardiovascular disease models^[^ ^41^ ^]^ including ischemic myocardial infarction,^[^ ^42^, ^43^ ^]^ cardiac hypertrophy,^[^ ^44^, ^45^ ^]^ chronic kidney failure induced heart failure^[^ ^46^ ^]^ and in cardiac endothelial cells in doxorubicin induced cardiotoxicity.^[^ ^47^ ^]^ Contrary to the notion of blocking myocardial apoptosis by using BAX and BAK inhibitors,^[^ ^48^, ^49^ ^]^ our results propose a model where doxorubicin drives mPTP opening (BAX, BAK, and VDAC) and releases mtDNA into the cytoplasm which triggers cGAS‐STING activation. In support, it has been demonstrated that by increasing SIRT1 expression to preserve mitochondrial integrity, the opening of mPTP and doxorubicin‐induced cardiotoxicity can be mitigated.^[^ ^50^, ^51^ ^]^ Moreover, mitochondria is necessary for irradiation inducing cellular senescence and cytoplasmic mtDNA triggers cellular senescence and senescence‐associated secretory phenotype (SASP).^[^ ^52^ ^]^ Our results also suggest that mtDNA‐cGAS‐STING signaling axis leads to expression of pro‐inflammatory cytokines in senescent cardiomyocytes which may explain why DIC are late onset in cancer surviving patients.

The mechanisms underlying doxorubicin‐induced cardiotoxicity are twofold. First, doxorubicin targets the mitochondria, disrupting the mitochondrial membrane structure, leading to a decrease in membrane potential and the generation of excessive reactive oxygen species (ROS), which induces apoptosis in hiPSC‐derived cardiomyocytes (hiPSC‐CMs). This apoptosis also requires the opening of mitochondrial permeability transition pores (MPTPs)^[^ ^10^ ^]^ and results in the leakage of mtDNA into the cytoplasm. This damage can be mitigated by approaches such as mitochondrial transplantation, which increase mitochondrial copy numbers. Second, the leakage of mtDNA into the cytoplasm activates the cGAS‐STING pathway, triggering the production of a large number of inflammatory factors that promote cellular senescence and further impair cardiomyocyte function. This second layer of damage can be suppressed through the knockout of STING or by overexpressing mitochondrial‐targeted DNase1.

Currently, cGAS‐STING inhibitors have been shown to be effective in alleviating myocardial infarction (MI) ^[^ ^53^ ^]^ and doxorubicin induced cardiotoxicity.^[^ ^54^ ^]^ However, this strategy raises the risk of silencing immune responses in the presence of an infection. Although mitochondrial transplantation holds promise as a preventative treatment to preserve cardiac function,^[^ ^10^ ^]^ single intramuscular injections not only poses secondary damage to the heart muscle but does not arrest cGAS‐STING activation. Alternatively, we provide proof‐of‐concept where overexpression of mitochondrial‐located DNase1 can stop mtDNA‐cGAS‐STING activation in its tracks. Although the idea of equipping transplanted mitochondria with mito‐DNase1 is appealing, more work and evidence is needed for this potential therapeutic approach.

## 4 Experimental Section

### Ethics

All animal studies were approved by the Institutional Animal Care and Use Committee (IACUC) at the University of Ninth People's Hospital, Shanghai Jiao Tong University School of Medicine (HKDL‐2018‐282), and performed according to the guidelines. Human studies were performed under Shanghai Children's Medical Centre, Shanghai Jiao Tong University School of Medicine no. SCMCIRB∼TKJB2023003. All protocols using human iPSC were reviewed and approved by the Ethics Review committee at Ninth People's Hospital, Shanghai Jiao Tong University School of Medicine (2018‐207‐K32).

### Doxorubicin‐Induced Heart Failure Mouse Model

Male C57BL/6 mice (6–8 weeks old; weighing 18–22 g) were obtained from Shanghai Jiesjie Laboratory Animal Co., LTD. Following a 1‐week acclimation period, the mice were randomly assigned to four groups (*n* = 5 per group): a control group (sham+Saline), a doxorubicin‐treated group (sham+Dox), a mitochondrial injection control group (mito+Saline) and a mitochondrial injection group with doxorubicin‐treated (mito+Dox). Mice in the Dox and mito+Dox groups received an intraperitoneal injection of doxorubicin (5 mg kg^−1^; MedChem Express) once a week for 4 weeks, while the Saline group received an equivalent volume of PBS. The mito+Dox group also received mitochondrial transplantation 1 day before each doxorubicin injection, with a total of 50 000 isolated mitochondria suspended in 80 µL (20 µL per injection site), as previously described.^[^ ^10^ ^]^

To obtain the cardiomyocyte‐specific Sting knockout mice, sting‐floxed mice were crossed with Myh6‐Cre mice as previously described. Age matched Sting^flox/flox^/Cre− and Sting^flox/flox^/Cre+ mice were used for the experiment. To overexpress mito‐DNase1, 2 weeks prior to Dox induction, mice received a 1E11 vg single i.v. injection of adeno‐associated serotype 9 (AAV9) virus carrying cardiac troponin T (cTnT) promoter‐driven mito‐DNase1 cDNA (AAV9‐mito‐DNase1). Empty AAV9‐cTnT served as vector control (AAV9‐Vector) (Genomeditech).

Transthoracic echocardiography was performed using the Vevo3100 High‐Resolution Imaging System (FUJIFILM VisualSonics) by an experienced investigator. Anesthesia was induced with 2% isoflurane and maintained with 0.5%–1.0% isoflurane to keep the heart rate between 410 and 450 beats per minute. M‐mode images from the left ventricular long‐axis view were obtained at the mid‐papillary muscle level. Echocardiographic data were analyzed using the VevoLAB Version 3.0 software package (FUJIFILM VisualSonics), and left ventricular ejection fraction and fractional shortening were measured as described.^[^ ^20^, ^55^ ^]^

At the sixth week (4 weekly Dox injections and 2 weeks rest), echocardiography was performed, and ventricular tissues were collected from euthanized mice. Hearts were subjected to Langendorff perfusion as previously described. Ventricular cardiomyocytes were seeded onto laminin‐coated (1:100 in dH_2_O; Sigma) 96‐well confocal glass‐bottom plates and used for MitoTracker Green (Thermo), MitoSox (Thermo), and TMRM (Thermo) live‐cell imaging on an Operetta CLS High Content Imaging System (PerkinElmer). The cells were imaged over time using the Operetta CLS High Content Imaging System (PerkinElmer).

### Blood Sample Collection and Mitochondria Copy Number Measurement

The sample was collected from a total of 11 patients that were scheduled to undergo doxorubicin treatment. All information was obtained from the electronic medical record system and outpatient or telephone follow‐up. Blood samples were freshly collected and extracted DNA immediately. All blood works were quantified by standard methods. DNA extraction was performed using a commercially available kit (Easy Pure Blood Genomic DNA Kit, Trans‐Gen Biotech, Beijing) following standard procedures. The average mitochondrial copy number of patient DNA samples were quantified by real‐time quantitative polymerase chain reaction (RT‐qPCR) in a LightCycler480 II Real‐Time PCR system (Roche Diagnostics International, Rot‐kreuz, Switzerland) using primers as previous described.^[^ ^56^ ^]^ All reactions were carried in triplicates.

### Cell Culture and Cardiac Differentiation

Human iPSCs were cultured on Matrigel (Corning) coated plates with Nutristem hiPSC XF medium (Biological Industries) and passaged every 2–3 days by performing a 1:6 dilution. At 70–90% confluency, hiPSCs differentiation was induced to generate beating CMs as described previously.^[^ ^57^ ^]^ Briefly, hiPSC were treated with 4–6 µM CHIR‐99021 (Selleck Chemicals) for 2 days, followed by a Wnt inhibitor IWR‐1 treatment (5 µM; Sigma) for another 2 days, in RPMI 1640 medium supplemented with B27 minus insulin (Thermo Fisher Scientific). On day 5, the medium was changed to fresh RPMI 1640 medium supplemented with B27 minus insulin for 2 days and switched to RPMI 1640 medium supplemented with B27 until day 10. hiPSC‐CMs were then purified using a metabolic selection medium which consisted of RPMI 1640 without glucose, B27 supplement (Life Technology), and 4 mM of sodium D‐Lactate (Sigma). The medium was changed every 2 days for the maintenance of cardiomyocytes.

### Doxorubicin Induced Apoptosis Titration Curve

On day 20, hiPSC‐CMs were seeded at ≈80% confluency in 96‐well plates. Doxorubicin at a concentration of 10 mM was serially diluted tenfold in the culture medium and added to the cells. After 24 h of incubation, the cells were fixed with 4% PFA and subjected to TUNEL staining using a TUNEL staining kit (Beyotime Biotechnology). Imaging and analysis were performed using the Operetta CLS High Content Imaging System (Perkin Elmer).

### Mitochondria Isolation

All mitochondria isolation from cells or tissue was performed as previously described.^[^ ^10^ ^]^ Briefly, fresh tissue or cells were collected and stored and washed once with PBS on ice. Tissues were cut up using scissors. Samples were transferred to Glass/Teflon Potter Elvehjem homogenizers with 3 mL ice‐cold Homogenizing Buffer (300 mM sucrose, 10 mM K‐HEPES, and 1 mM K‐EGTA, pH adjusted to 7.2, stored at 4 °C), homogenized, and transferred to centrifuge tube. We added 250 mL Subtilisin A Solution (1 mg Subtilisin A in 250 mL homogenizing buffer) to the homogenate, mixed by inversion, and incubated on ice for 10 min. Homogenate was filtered into a 50 mL conical centrifuge tube using a 40‐mm mesh on ice and filter and 250 mL freshly prepared bovine serum albumin (BSA) Stock Solution (5 mg BSA in 250 mL homogenizing buffer) was added and mixed by inversion. Repeat homogenate filtration into a 50‐mL conical centrifuge tube using a 10‐mm filter on ice. Transfer the filtrate to two pre‐chilled 1.5‐mL microfuge tubes and centrifuge at 9000 g for 10 min at 4 °C. Remove supernatant and re‐suspend in 1 mL homogenizing buffer, and centrifuge at 7000 g for 10 min at 4 °C. Remove the supernatant, resuspend in PBS, and store at 4 °C and use within 2 h.

### Mitochondria Clearance

YFP‐Parkin system was used to delete mitochondria as previously described.^[^ ^56^ ^]^ YFP‐Parkin was conducted into lentivirus with two package plasmids using lipo‐transfect (Yeasen). After infecting HL‐1 cells and hiPSC‐CMs with YFP‐Parkin lentivirus for 72 h, the cells were treated with a culture medium containing 6.25 µM CCCP (Sigma) every 12 h for a total of 48 h. Following this treatment, mitochondrial clearance efficiency was assessed.

### Cardiomyocyte Contractility Assay

The contractility of adult mouse cardiomyocytes (AMCMs) was assessed using an IonOptix System (IonOptix). Briefly, AMCMs were isolated via the Langendorff method and seeded onto laminin‐coated imaging dishes (Sigma‐Aldrich) in DMEM supplemented with 10% FBS. The cells were incubated at 37 °C for 30 min. AMCMs were then field stimulated at 10 V with a frequency of 1 Hz, and sarcomere length changes were simultaneously recorded and calculated.

For hiPSC‐CMs, spontaneous contractions were recorded using an Olympus IX83 inverted microscope (Olympus) at 50 frames per second (fps). Contraction speeds were determined using a MATLAB‐based motion‐tracking software (MathWorks), as previously described.^[^ ^58^ ^]^

### Oxygen Consumption Measurements

Oxygen consumption rates (OCRs) of hiPSC‐CM were measured using a Seahorse XFe96 Extracellular Flux Analyzer in conjunction with the Seahorse Cell Mito Stress Test Kit (Agilent). hiPSC‐CM were seeded onto Seahorse cell culture plates and treated according to the manual. Prior to measurement, the culture medium was replaced with Seahorse XF RPMI supplemented with 5 µM glucose, 1 µM pyruvate, and 10 µM glutamine, and cells were incubated for 1 h at 37 °C in a CO_2_‐free incubator. OCRs were then measured under basal conditions and in response to sequential treatments with oligomycin (1.5 µM), FCCP (1 µM), and Rotenone/Antimycin A (0.5 µM). The rates were normalized to total cell number, which was determined by Hoechst 33 342 staining and quantified at the end of the Seahorse experiment.

### Protein Extraction and Western Blot

Protein extraction from adult mouse cardiomyocytes (AMCMs) and hiPSC‐CM was performed at 4 °C using radioimmunoprecipitation assay (RIPA) buffer supplemented with a protease and phosphatase inhibitor cocktail. Following cell lysis, samples were centrifuged at 12 000 rpm for 15 min at 4 °C, and the supernatant containing protein lysates was transferred to a fresh Eppendorf tube. Protein concentrations were determined using the bicinchoninic acid (BCA) assay (Beyotime Biotechnology). Digital imaging and quantification were conducted using the Odyssey Infrared Imaging System (LI‐COR).

### RNA Preparation and Quantitative Real‐Time PCR

Total RNA was extracted using TRIzol reagent (Invitrogen) and reverse‐transcribed according to the manufacturer's instructions using the HiScriptII Reverse Transcriptase kit (Vazyme). Quantitative real‐time PCR (qPCR) was performed in triplicate using an Applied Biosystems 6Flex system (Applied Biosystems) with SYBR Green Master Mix (Bimake). Gene expression levels were calculated using the 2^−∆∆Ct^ method, normalized to Gapdh mRNA, and expressed as fold changes. The sequences of all primers used are listed in Table S2 (Supporting Information).

### Immunofluorescence Staining

Heart sections were deparaffinized and rehydrated through xylene and ethanol gradients, followed by PBS washes. Antigen retrieval was carried out in Tris‐EDTA solution for 20 min at 95 °C. For hiPSC‐CM, cells were rinsed with PBS, fixed with 4% paraformaldehyde for 20 min at room temperature, then blocked with PBS containing 10% goat serum (Beyotime Biotechnology) and 0.3% Triton‐100 (Sigma‐Aldrich) for 1 h at 37 °C. Sections and hiPSC‐CMs were incubated overnight at 4 °C with primary antibodies (Table S4, Supporting Information), followed by three PBS washes. Alexa Fluor‐conjugated secondary antibodies (Table S4, Supporting Information) were applied for 1 h at room temperature, and nuclei were counterstained with 4′,6‐diamidino‐2‐phenylindole (DAPI) for 10 min. Imaging was performed using a Zeiss LSM880 confocal microscope, with analysis conducted in ZEN software (Zeiss).

### Transmission Electron Microscopy

For transmission electron microscopy, freshly excised heart tissues were immediately fixed in 2.5% glutaraldehyde at 4 °C overnight. Samples were washed three times with 0.1 M cacodylate buffer, post‐fixed in 1% osmium tetroxide for 1 h, and washed three more times with PB buffer. Dehydration was performed using ethanol gradients, followed by incubation in acetone, and embedding in ethoxyline resin. Ultrathin sections were mounted on copper grids, and images were captured using a FEI Tecnai G2 Spirit 120 kV electron microscope (FEI Italia).

### Statistical Analysis

Student's t test was used for two‐group continuous variable comparisons, whereas the Mann‐Whitney U test was used for categorical variable comparisons. Analysis of variance (ANOVA) was used for multiple group variable comparisons. All tests were two way, and a P value of <0.05 was considered significant. Data visualization in all figures, as well as statistical analysis, was accomplished by GraphPad Prism 9.0.0. Data are shown as means ± SEM.

## Conflict of Interest

The authors declare no conflict of interest.

## Author Contributions

W.X., B.L., and J.P. contributed equally to this work. W.X., A.C.Y.C., and M.L. conceived and designed this project. J.Z., L.F., Y.Z., and H.Y. provided reagents. W.X., B.L., J.P., D.L., and X.W. performed the in vivo experiments. W.X. and B.L. performed the in vitro experiments. W.X. analyzed data and drafted the manuscript. A.C.Y.C., M.L., and J.Z. revised the initial draft. All authors provided critical discussions, proof‐read the draft, and gave final approval of the manuscript.

## Supporting information



Supporting Information

## Data Availability

All data are available in the main text or the supplementary materials. The data that support the findings of this study are available from the corresponding author upon reasonable request.
