# Supplementary material for: Mitochondrial Amount Determines Doxorubicin‐Induced Cardiotoxicity in Cardiomyocytes
Source: Adv Sci (Weinh). 2025 Feb 7;12(12):2412017. doi: 10.1002/advs.202412017 (PMC11948046; doi:10.1002/advs.202412017)
Supplement: Supplementary file 1 — Supporting Information [file ADVS-12-2412017-s001.docx]

Supporting Information

**Mitochondrial Amount Determines Doxorubicin-Induced Cardiotoxicity**

**in Cardiomyocytes**

*Weiyao Xiong^1,2#^, Bin Li^1,2^**^#^, Jianan Pan^2#^, Dongjiu Li^2^, Haihua Yuan^3^, Xin Wan^1,2^, Yanjie Zhang^3^, Lijun Fu^4^, Junfeng Zhang^1^*, Ming Lei^2^*, Alex Chia Yu Chang^1,2^**

^1^ Department of Cardiology, Shanghai Ninth People’s Hospital, Shanghai Jiao Tong University School of Medicine, Shanghai, China

^2^ Shanghai Institute of Precision Medicine, Shanghai Ninth People’s Hospital, Shanghai Jiao Tong University School of Medicine, Shanghai, China

^3^ Department of Oncology, Shanghai Ninth People’s Hospital, Shanghai Jiao Tong University School of Medicine, Shanghai, China

^4^ Department of Cardiology, Shanghai Children's Medical Centre, Shanghai Jiao Tong University School of Medicine, Shanghai, China

^#^These authors contributed equally

**Table S1. The baseline characteristics of the included patients.**

| **Patient ID** | **Sex** | **Age** | **Height(cm)** | **Weight before medication(kg)** | **Weight after medication(kg)** | **Total accumulation(mg)** |
| --- | --- | --- | --- | --- | --- | --- |
| 1 | Male | 56 | 173 | 87 | 88 | 40 |
| 2 | Female | 67 | 150 | 67.5 | 54 | 350 |
| 3 | Female | 50 | 160 | 70 | 62 | 480 |
| 4 | Male | 67 | 163 | 63 | 65 | 40 |
| 5 | Female | 58 | 163 | 59 | 59 | 150 |
| 6 | Female | 58 | 160 | 61 | 65 | 420 |
| 7 | Male | 74 | 180 | 76 | 75 | 200 |
| 8 | Female | 53 | 158 | 63 | 61 | 260 |
| 9 | Female | 43 | 162 | 57 | 58 | 130 |
| 10 | Male | 58 | 171 | 85 | 85 | 160 |
| 11 | Female | 68 | 155 | 58 | 55 | 150 |

**Table S2. Real-Time quantitative PCR Primers.**

| **Primer** | **Forword** | **Reverse** |
| --- | --- | --- |
| *Human cGAS* | AAGGATAGCCGCCATGTTTCT | TGGCTTTCAGCAAAAGTTAGG |
| *Human Sting* | AGCATTACAACAACCTGCTACG | GTTGGGGTCAGCCATACTCAG |
| *Mouse cGAS* | GTTCAAACACAAGAAATGCACTG | GCTGACGGAGTACACAATCCT |
| *Mouse Sting* | TGAAAGGCTCTTCATTGTCTCTT | TGGCATCTTCTGCTTCCTAGA |
| *Mouse Tnf* | CCAAATGGCCTCCCTCTCAT | TGGTGGTTTGCTACGACGTG |
| *Mouse Isg15* | CTAGAGCTAGAGCCTGCAG | AGTTAGTCACGGACACCAG |
| *Human Ifnb1* | TGTCGCCTACTACCTGTTGTGC | AACTGCAACCTTTCGAAGCC |
| *Human Ifit1* | CAAGGCAGGTTTCTGAGGAG | GACCTGGTCACCATCAGCAT |
| *Human IL-6* | GGTACATCCTCGACGGCATCT | GTGCCTCTTTGCTGCTTTCAC |
| *Mouse GAPDH* | GGAGAGTGTTTCCTCGTCCC | ATGAAGGGGTCGTTGATGGC |
| *Human GAPDH* | CCTCAACGACCACTTTGTCA | TTACTCCTTGGAGGCCATGT |
| *MTRNR2* | CGAAAGGACAAGAGAAATAAGG | CTGTAAAGTTTTAAGTTTTATGCG |
| *β-Globin* | CAACTTCATCCACGTTCACC | GAAGAGCCAAGGACAGGTAC |

**Table S3 Sequences of gene-shRNA.**

| **shRNA** | **Sequence** |
| --- | --- |
| sh*Bax* | CCAGCTCTGAGCAGATCATGA |
| sh*Bak* | CCCACGGCAGAGAATGCCTAT |
| sh*VDAC1* | GCTATGGATTTGGCTTAATAA |
| sh*VDAC3* | CGTCATGTTAGAGGAGACGAT |
| NC | UUCUCCGAACGUGUCACGUTT |

**Table S4 Antibodies.**

| **Antibodies** | **Cat. No. and Company** |
| --- | --- |
| cTnT | Ab8295, abcam |
| TOM 20 | Cat No. 11802, Proteintech |
| dsDNA | Ab273137, abcam |
| cGAS | sc-515777, Santa Cruz |
| STING | Cat No. 19851, Proteintech |
| GAPDH | Cat No. 60004, Proteintech |
| DNase1 | sc-376207, Santa Cruz |
| P21 | 2947, CST |
| P53 | Cat No. 10442, Proteintech |
| P16 | 18769, CST |
| TBK1 | 3504, CST |
| p-TBK1 | 5483, CST |
| Anti-mouse IgG (H+L) (DyLight™ 800 4X PEG Conjugate) | 5257, CST |
| Anti-rabbit IgG (H+L) (DyLight™ 800 4X PEG Conjugate) | 5151, CST |
| Anti-rabbit IgG (H+L), F(ab')2 Fragment (Alexa Fluor® 594 Conjugate) | 8889, CST |
| Anti-mouse IgG (H+L), F(ab')2 Fragment (Alexa Fluor® 488 Conjugate) | 4408, CST |
| Anti-mouse IgG (H+L), F(ab')2 Fragment (Alexa Fluor® 594 Conjugate) | 8890, CST |


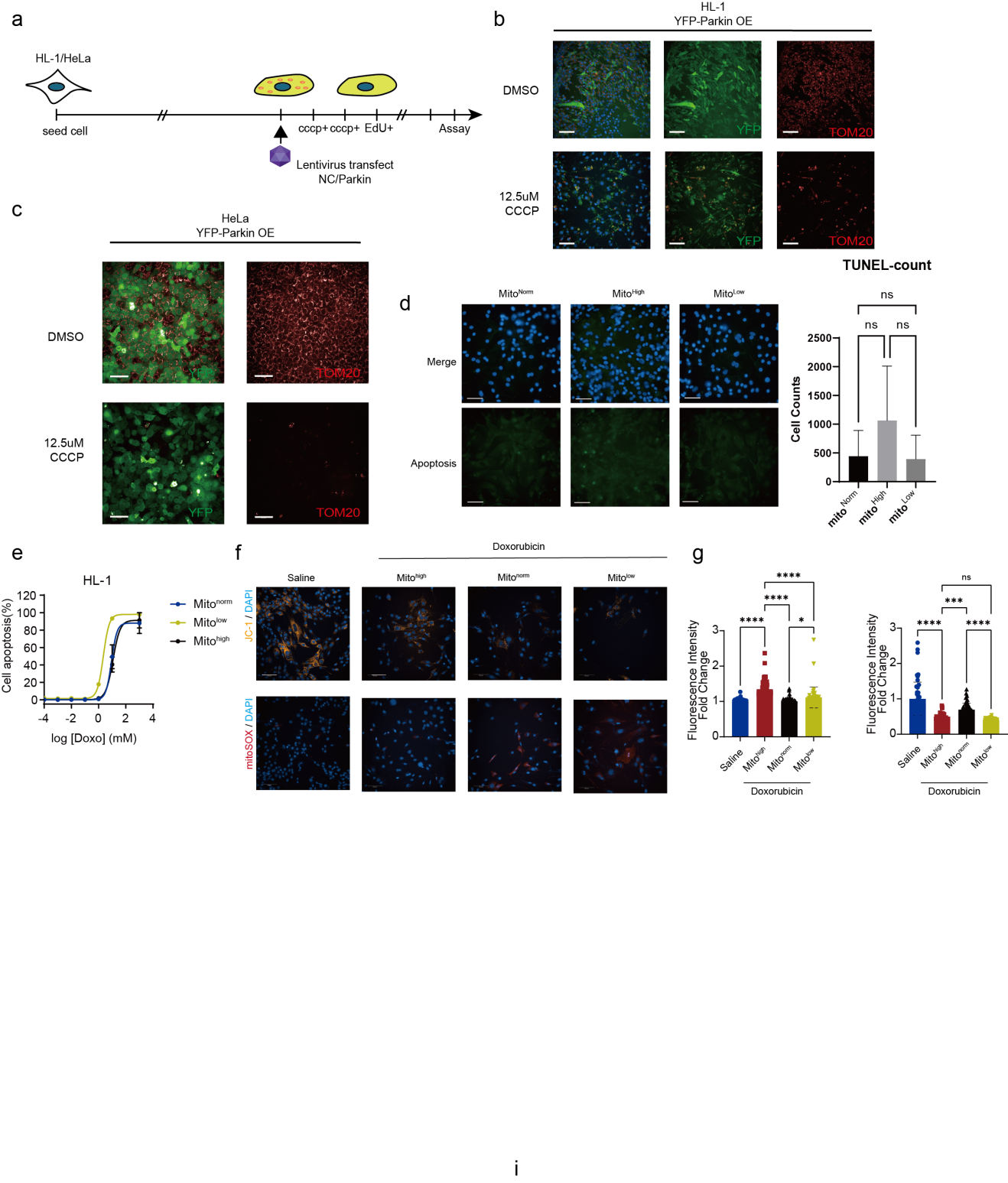


**Figure S1. Detecting the correlation of mitochondrial copy number and doxorubicin tolerance in HL-1 and HeLa cells.**

a) Illustration of the experimental method for constructing cell lines with reduced mitochondria. b) Immunofluorescence shows successful construction of cells with reduced mitochondria using the YFP-Parkin system with 12.5 μM CCCP for 48 h in HL-1 cells, green indicating YFP and red indicating mitochondria (TOM20), scale bar = 100 μm. c) Immunofluorescence shows successful construction of cells with reduced mitochondria using the YFP-Parkin system with 12.5 μM CCCP for 48 h in HeLa cells, green indicating YFP and red indicating mitochondria (TOM20), scale bar = 100 μm. d) TUNEL staining of mito^high^, mito^norm^, and mito^low^ hiPSC-CM shows no background apoptosis in mitochondrial clearance and mitochondria transplant, scale bar = 100 μm, n = 3, quantification values are expressed as mean ± SEM. e) Apoptosis curves show that HL-1 cells with fewer mitochondrial DNA copy numbers have lower doxorubicin tolerance, n = 3, quantification values are expressed as mean ± SEM, TUNEL assay after Doxorubicin treated 24 h. f,g) JC-1 and mito-SOX staining shows that after doxorubicin treatment, HL-1 cells with more mitochondria maintain mitochondrial membrane potential (orange), whereas cells with fewer mitochondria exhibit decreased membrane potential, doxorubicin 1 μM, 24 h, scale bar = 100 μm, n = 30, quantification values are expressed as mean ± SEM.


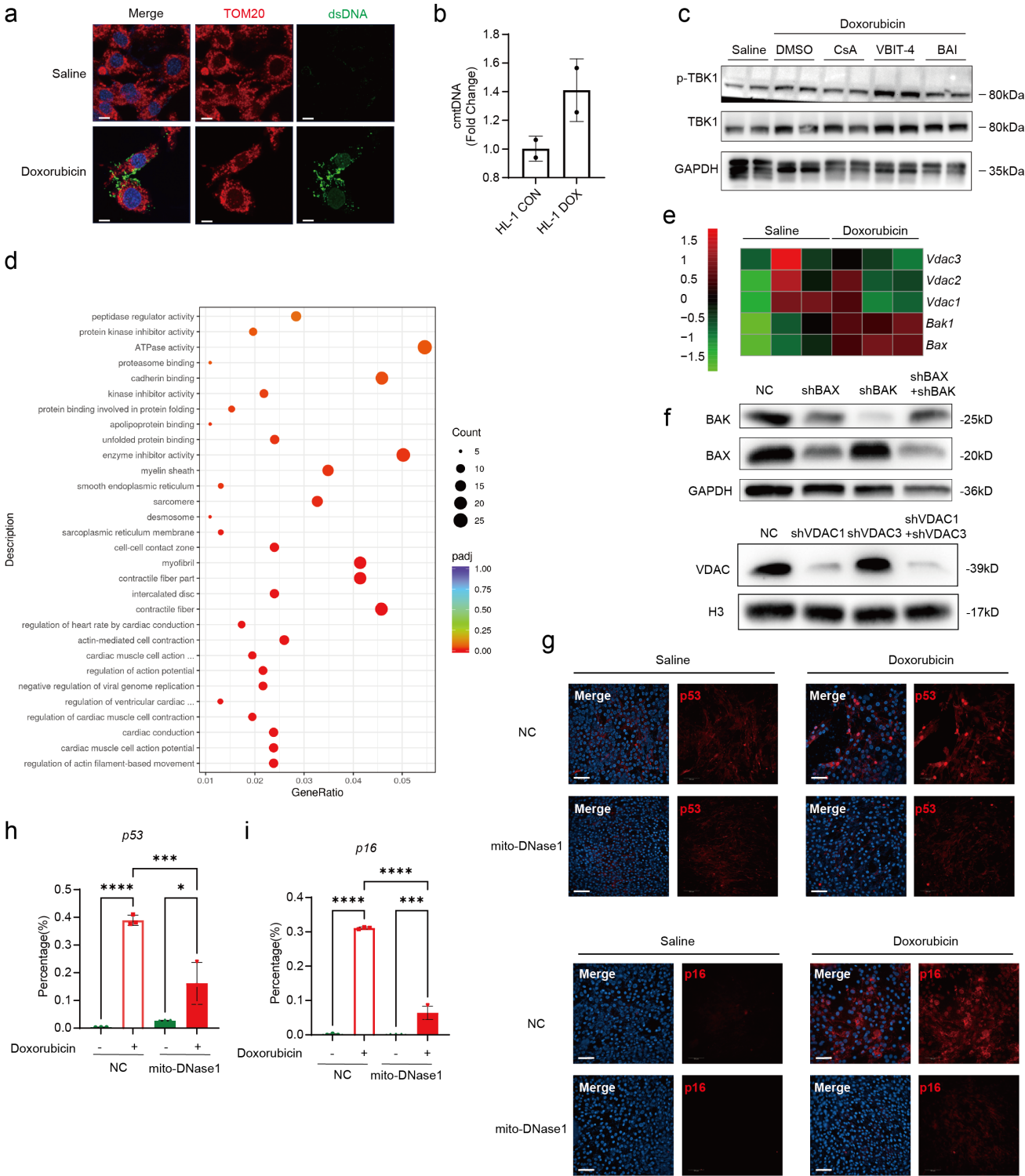


**Figure S2. Doxorubicin opens mPTP activated mtDNA-cGAS-STING pathway accelerate senescence.**

a) Confocal microscopy shows that doxorubicin induces mtDNA leakage into the cytoplasm in HL-1 cells, green representing dsDNA, red representing mitochondria (TOM20), scale bar = 10 μm. b) qPCR detected the levels of cytoplasmic mtDNA (cmtDNA) leakage in HL-1 cells under different treatments, n = 2. c) Immunoblotting shows the activation of the cGAS-STING pathway in HL-1 cells treated with doxorubicin (1 μM, 24 h) with and without the addition of BAX/BAK inhibitors (BAI1, 1 μM, Pre-treated 1h) and VDAC inhibitors (VBIT-4, 15 μM, Pre-treated 1h), n = 3. d) GO enrichment analysis shows downregulation of mitochondrial transcription-related pathways in purified AMCMs after doxorubicin treatment, n = 3. e) Bulk RNA-seq shows increased expression of mitochondrial membrane channel proteins after doxorubicin treatment in purified AMCMs, n = 3. f) Immunoblotting shows the knockdown efficiency of shBAX, shBAK, shVDAC1, and shVDAC3 in hiPSC-CMs. g,h,i) Immunofluorescence staining shows a significant reduction of p53, p16 positive cells in hiPSC-CMs with mitochondria-targeted DNase1 OE following doxorubicin treatment, scale bar = 100 μm, n = 3, quantification values are expressed as mean ± SEM.


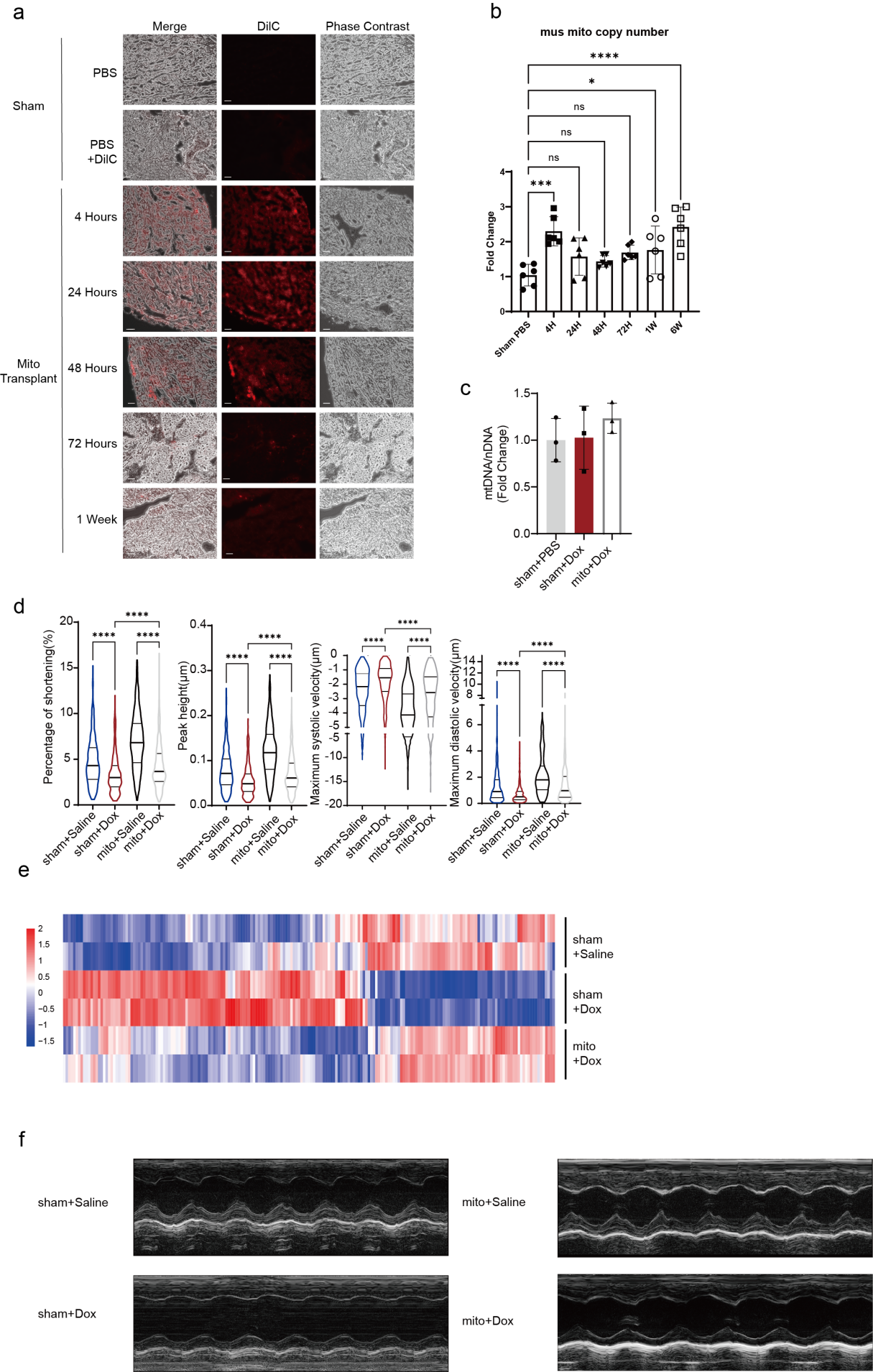


**Figure S3. Mitochondrial transplantation can rescue DIC.**

a) DilC-positive mitochondrial signals were observed in 8 μm-thick frozen sections of mouse hearts at 4 hours, 24 hours, 48 hours, 72 hours, and 1 week post-injection. scale bar = 50 μm, red indicate DilC positive mitochondria, phase-contrast are used in observe murine heart section. b) RT-qPCR shows increase in mouse mtDNA copy number, n = 6, quantification values are expressed as mean ± SEM. c) qPCR shows no significant change in mitochondrial copy number in myocardial cells treated with doxorubicin after *in situ* mitochondrial transplantation, n = 3, quantification values are expressed as mean ± SEM. d) Ionoptix detection of isolated adult mouse cardiomyocytes under simulated physiological beating conditions shows that *in situ* transplantation of cardiac mitochondria alleviates doxorubicin-induced cardiotoxicity, n = 100, quantification values are expressed as mean ± SEM. e) Bulk RNA-seq reveals differentially expressed genes in myocardial cells treated with doxorubicin after *in situ* mitochondrial transplantation, n = 2. f) Echocardiography shows that *in situ* transplantation of cardiac mitochondria partially alleviates doxorubicin-induced cardiac dysfunction.


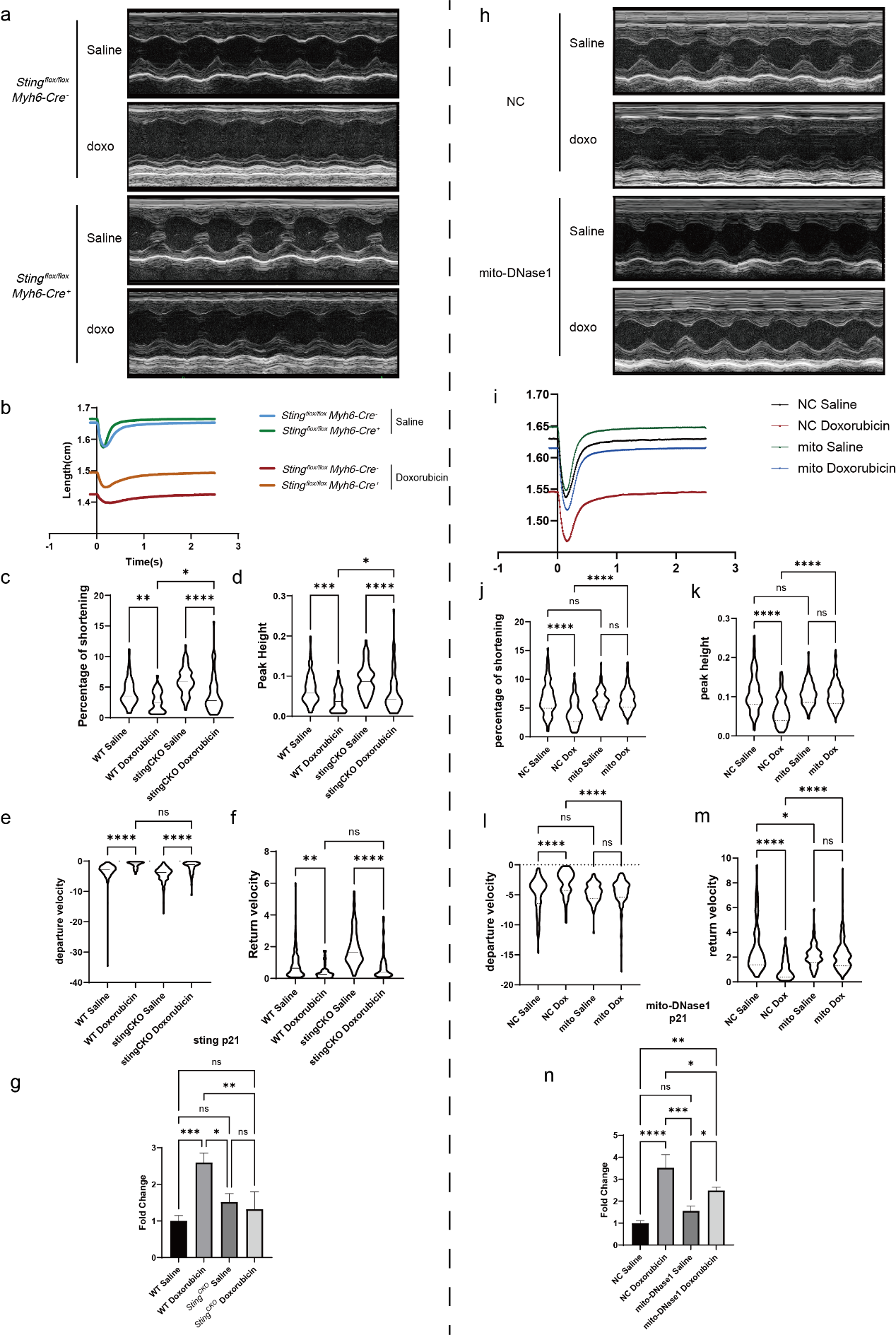
 **Figure S4. Sting^CKO^ and over expressed mito-DNase1 can rescue DIC.**

a) Echocardiography shows that myocardial specific STING knockout partially alleviates doxorubicin-induced cardiac dysfunction. b,c,d,e,f) Ionoptix detection of isolated adult mouse cardiomyocytes under simulated physiological beating conditions shows that myocardial specific STING knockout alleviates doxorubicin-induced cardiotoxicity, n = 100, quantification values are expressed as mean ± SEM. h) Echocardiography shows that myocardial over expressing mitochondria-targeted DNase1 partially alleviates doxorubicin-induced cardiac dysfunction. i,j,k,l,m) Ionoptix detection of isolated adult mouse cardiomyocytes under simulated physiological beating conditions shows that over expressing mitochondria-targeted DNase1 alleviates doxorubicin-induced cardiotoxicity, n = 100, quantification values are expressed as mean ± SEM. g,n) Quantitative analysis demonstrates that p21 expression is significantly suppressed in Sting^CKO^ mice and mito-DNase1 overexpression mice following doxorubicin treatment.
